# Examining the Relationship between Cellphone Use Behavior, Perceived Exercise Benefit and Physical Exercise Level among University Students in Taiwan

**DOI:** 10.3390/healthcare8040556

**Published:** 2020-12-11

**Authors:** Mei-Ling Lin, Wen-Yi Wang, Chun-Chin Liao, Yu-Jy Luo, Chun-Chieh Kao

**Affiliations:** 1 Office of Physical Education, Mackay Junior College of Medicine, Nursing & Management, Taipei City 111, Taiwan; s091@mail.mkc.edu.tw; 2 Graduate Institute of Sport Pedagogy, University of Taipei, Taipei City 111, Taiwan; wenyiwang1225@gmail.com; 3 Office of Physical Education, Ming Chuan University, Taoyuan City 333, Taiwan; luck@mail.mcu.edu.tw (C.-C.L.); anitaluo@mail.mcu.edu.tw (Y.-J.L.)

**Keywords:** physical education teachers, health benefits, cellphone usage frequency, moderating effect, physical activity

## Abstract

This study investigated how perceived exercise benefit affects the relationship between cellphone usage and physical activity level. This cross-sectional study performed a survey of Taiwanese university students selected using cluster sampling. A total of 975 students were recruited (male = 367, female = 608, average age = 20.10 ± 1.42). Data were analyzed using descriptive statistics, correlation analysis, and hierarchical regression. The results show that cellphone usage was negatively correlated with physical activity level, whereas perceived exercise benefit was negatively correlated with cellphone usage and positively correlated with physical activity level. In hierarchical regression, the main effects of cellphone usage and perceived exercise benefit explained 22% of the variance in physical activity level. After controlling for the main effect, the interaction term accounted for an additional 1% of the variance. Cellphone usage and perceived exercise benefit thus had significant power to explain physical activity level. The results of this study reveal a novel phenomenon—that students who perceived the benefits of exercise to be greater are more physically active.

## 1. Introduction

Physical activity plays a crucial role in the World Health Organization’s global action plan for the prevention and control of noncommunicable diseases 2013–2020 [1]. Lack of physical activity causes more than five million premature deaths annually and is one of the most critical factors in the global disease burden [2]. The chronic diseases and other negative health consequences caused by lack of physical activity are quickly becoming pressing public health concerns [3,4]. Regular exercise has myriad health benefits such as an improved sense of wellbeing, and reduced depression, anxiety or stress (74%) [5]. Nevertheless, more than half of the world’s youth population do not perform enough physical activity to reap these benefits [6,7]. In recent decades, the level of physical activity has declined considerably [8]. Several studies examining people’s understanding of the existing physical activity guidelines and benefits of physical activity revealed the following: 40% of adults in Iran had a low level of physical activity, with approximately 15% (4.7 million) doing no physical activity at all [9], and in Canada, 85% of adults did not achieve the target of 150 min of moderate to vigorous physical activity per week [4]. Another study examining the public’s understanding of physical activity guidelines revealed that only 36% of the American population understood the physical activity guidelines and the benefits of physical activity [10]; in the UK, only 15% of the population could correctly answer questions related to the physical activity guidelines [11] and in China, only 4% of the university population had accurate understanding of the benefits of physical activity [5]. Moreover, according to a survey that was conducted in 20 countries/territory, adults in Taiwan spent little time exercising but spend longer time sitting. Within these 20 countries/territory, Taiwanese adults spend more than 9 h sitting per day, which is second only to the result of Japanese people of 34.9%. In addition, 18.6% of Taiwanese people spend 6 to 9 h sitting per day [12]. The university stage is usually considered as a phase when people reach adulthood, and there is evidence of negative changes in physical activity and sedentary behavior during this period [13]. Some studies investigated and tracked the physical activity of individuals from adolescence to adulthood and suggested that university may be a period when students cultivate their lifelong physical activities and sedentary behavior [13]. The decline in physical activity has led to various problems in student populations, including being overweight, obesity and other health problems [12,14,15]. As physical activity levels have declined, prolonged sitting has become more prevalent, and physical inactivity is now the fourth leading risk factor for global mortality (6% of deaths globally) [16]. In summary, physical inactivity has become one of the largest public health challenges of this age [17].

Studies have discovered a direct negative relationship between cellphone usage and physical activity [18]. Using a cellphone while exercising results in the exercise being performed at lower intensity, weakening the effects of the exercise [19]. Smartphones are no longer tools only for two-way communication. For modern university students, a cellphone-centered lifestyle is slowly becoming commonplace [20]. Such a lifestyle not only reduces students’ physical activity levels but also has various negative health consequences [21,22,23]. The results of an Asia cellphone usage survey published by Yahoo in 2014 revealed that, on average, Taiwanese people spend more than 3 h on their cellphone daily, and cellphone reliance is as prevalent as 81%, which makes Taiwan the country/territory in Asia with the most excessive cellphone usage [24]. More than 71% of university students spend more than 4 h on their cellphone each day [25]. University is typically considered the transition period between adolescence and adulthood. Cellphone addiction is common among university students and affects their physical and mental health [21,26]. Negatively correlated with academic performance [27,28], cellphone addiction can also result in low self-esteem, increased prolonged sitting and decreased social behaviors [21] in this population. Use of cellphones has altered people’s daily living habits, social behaviors, emancipative values, familial relationships and social interactions [29]. Given the severity of this problem, further studies must be conducted to confirm the potential relationships between cellphone usage, physical activity level and perceived exercise benefit.

Establishing a positive environment that can influence students’ physical activity levels, and their perceptions of the benefits of physical activity, is one of the main functions of physical education [5,30]. The role of the school physical education curriculum in promoting healthy lifestyles is publicly acknowledged [31], and schools are critical environments in which young people can increase their level of physical activity [32,33]. Schools play a potentially essential role in establishing healthy lifestyles and regular exercise habits [34,35]. A school-based physical education curriculum is ideal for making students more physically active because students spend much more time at school than at any other place other than their home [34]. One study discovered that physical education plays a critical role in the academic cognition of students [36]. In short, physical education activities are not only extracurricular activities and educators should improve their dissemination of information related to the importance of an active lifestyle and health promotion in physical education [34]. The value of physical education is not only in increasing physical activity in students; physical education curricula should also expand students’ understanding of the potential effects of physical activity on health. Therefore, other than cultivating good health and regularly exposing students to various sporting activities, physical education should provide students with an understanding of the value of physical activity and the benefits of adopting a healthy lifestyle [37]. Through physical education teaching and learning, educators can equip students with sufficient health knowledge and increase the level of physical activity in students.

In the literature, studies assessing the relationship between physical activity level and perceived health benefits are few [38]. In addition, no direct evidence has yet been obtained that cellphone usage and perceived exercise benefit affect physical activity level. The main purpose of this research is to evaluate how perceived exercise benefit can affect the relationship between cellphone usage and physical activity level using the perceived exercise benefit scale and International Physical Activity Questionnaire. The research results may help to understand the relationship between cellphone usage and sports training level, and also the moderating effects of perceived exercise benefit. Therefore, a unique contribution of this study is to support and expand this novel research area.

## 2. Materials and Methods

### 2.1. Participants and Procedures

This study adopted a cross-sectional research design. A survey was performed in spring 2019 to examine the relationships between cellphone usage frequency (divided into five categories (under 2 h, 2 to under 4 h, 4 to under 6 h, 6 to under 8 h and above 8 h)), perceived exercise benefit, and physical activity level. Cluster sampling was employed, and the sampled population was the university students of Taiwan. The first stage of sampling was conducted according to geographical region, with Taiwan divided into the north, center, south and east. In the second stage of sampling, the schools in each region were randomly sampled. The sample size was estimated based on the determination of a confidence interval (CI) at 95%, statistical power of 80% and α of 0.05. Based on these, it was planned to recruit at least 120 students randomly from each school. A total of 1159 physical education students were recruited (men = 367, women = 608, average age = 20.10 ± 1.42 years). Responses were incomplete for 146 respondents, who were excluded from the analysis. Additionally, 38 potential respondents refused to participate in the study. The final sample size was thus 975.

### 2.2. Ethical Approval

All procedures, including the informed consent and the recruitment of participants, were reviewed and approved by the Thai Clinical Trials Registry Committee (TCTR), and were satisfactory for all items of the trial registration data set required by the World Health Organization. The identification number for the clinical trial in this study is TCTR20190507005.

### 2.3. Research Tools

#### 2.3.1. Perceived Exercise Benefit Scale (PEBS)

A perceived exercise benefit scale was designed to assess an individual’s perceptions of the benefits that may be gained from physical activity. The scale was amended from the scale developed by Chang, Li and Sheu [39], which contains 10 items (e.g., “I think that doing physical activity improves my cardiopulmonary function and…”), and the participants indicated their level of agreement with the statements by ticking relevant answer boxes. The scale was a five point scale (1 = strongly disagree; 2 = disagree; 3 = neutral; 4 = agree; 5 = strongly agree). The Cronbach’s α coefficient of the internal table was 0.90, the retest reliability was 0.89, and the explainable variance was 58.77%. The score range was between 5 and 50. The higher the total score, the higher the degree of perceived exercise benefit. The scale showed acceptable fit (χ² = 52.535, RMSEA = 0.07, SRMR = 0.022, CFI = 0.986, GFI = 0.986, AGFI = 0.968, IFI = 0.986, NNFI = 0.976). The average variance extracted (AVE) of the latent variable was 0.514, with a composite reliability (CR) of 0.861. Thus, we concluded that the perceived exercise benefit scale was psychometrically sound, with adequate reliability and validity.

#### 2.3.2. Taiwan Version of the International Physical Activity Questionnaire—Short Form

The Taiwan version of the International Physical Activity Questionnaire—short form (IPAQ-sf), published by the Ministry of Health and Welfare of the Taiwan Executive Yuan, was employed to determine the students’ physical activity levels. This Chinese version of the IPAQ-sf was developed from Liou [40]. With a content validity index of 0.994, both the English and Chinese versions of the IPAQ-sf could be considered to have favorable content validity. The intraclass correlation coefficient between the Chinese and English versions of the IPAQ-sf was discovered to be 0.945 [41]. On the basis of the tertile distribution of their IPAQ-sf scores, the participants were assigned to three groups: those with low (<33rd percentile), medium (33rd–66th percentile) and high (>66th percentile) scores.

### 2.4. Data Analysis

All data were analyzed using SPSS software (version 22 for Windows, IBM, Armonk, NY, USA). Three data analyses were conducted: (1) descriptive statistics: used to analyze the distribution of the sample’s demographic characteristics and study variables; (2) correlation analysis employed to analyze the correlations between cellphone usage, perceived exercise benefit, and physical activity and (3) hierarchical regression used to assess the moderating effect of perceived exercise benefit on the relationship between cellphone usage and physical activity. Through the interaction of the independent variable and moderating variable, the researchers determined whether a moderating effect existed. The level of significance for all data analyses was set at α < 0.05.

## 3. Results

### 3.1. Descriptive Statistics and Correlation Analysis

All 975 participants of this study were university students (male = 367, female = 608, average age = 20.10 ± 1.42). The most common daily cellphone usage was 4 to under 6 h daily (*n* = 333, 34.15%), and the least common daily usage was usage for less than 2 h daily (*n* = 34, 3.49%). The majority of the participants did sufficient physical activity (*n* = 533, 54.67%), with those doing a high level of physical activity being in the minority (*n* = 182, 18.67%). The mean value on the perceived exercise benefit scale was 38.88 (standard deviation = 7.155). The coefficients of correlation between the variables are displayed in Table 1. The correlation analysis indicated that cellphone usage was negatively correlated with physical activity level (*r* = −0.23). Perceived exercise benefit was discovered to be negatively correlated with cellphone usage (*r* = −0.10). Thus, the greater the cellphone usage, the lower the participants’ perceived exercise benefit. Lastly, the analysis revealed that perceived exercise benefit was positively correlated with physical activity level (*r* = 0.43), the higher the level of perceived exercise benefit, the higher the participants’ physical activity levels.

### 3.2. Relationships between Cellphone Usage, Perceived Exercise Benefit and Physical Activity Level

Hierarchical regression was performed to examine the effects of cellphone usage and perceived exercise benefit on physical activity level. The results of this analysis are displayed in Table 2. The main effects of cellphone usage and perceived exercise benefit explained 22% of the variance in physical activity level (*F* (2, 972) = 134.77, *p* < 0.001). After controlling for the main effect, the interaction term accounted for an additional 1% of the variance (F (1, 971) = 6.03, *p* < 0.05).

**Figure 1:**
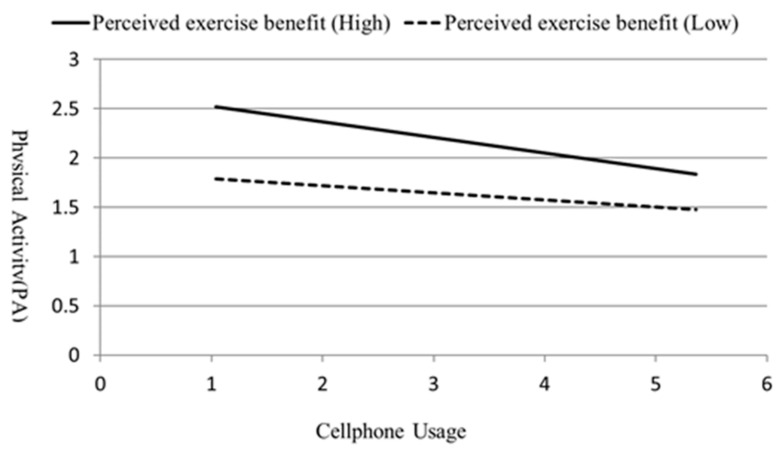
Simple slope interaction plot.

In terms of the main effects, cellphone usage had significant power to explain physical activity level (β = −0.19, *p* < 0.001), indicating that physical activity level was lower when cellphone usage was greater. Additionally, perceived exercise benefit had significant explanatory power over physical activity level (β = 0.41, *p* < 0.001). This result indicated that the participants who perceived more benefits of exercise were more physically active. Lastly, the interaction term of cellphone usage and perceived exercise benefit was discovered to have significant explanatory power for physical activity (β = −0.07, *p* < 0.05). The results of simple slope analysis indicated that when the level of perceived exercise benefit was high, cellphone usage had significant exploratory power for physical activity level (β = −0.16, *p* < 0.001; Figure 1), compared with the figure when the level of perceived exercise benefit was low (β = −0.07, *p* < 0.001; Figure 1). The regression coefficients revealed that the explanatory power of cellphone usage over physical activity level was higher for the high-perceived-exercise-benefit group than for the low-perceived-exercise-benefit group.

## 4. Discussion

The current study revealed significant correlations between cellphone usage frequency, physical activity level and perceived exercise benefit. Cellphone usage was discovered to be negatively correlated with physical activity level (*r* = −0.23) and perceived exercise benefit (*r* = −0.10). Thus, the greater the cellphone usage, the lower the participants’ physical activity levels and perceived exercise benefit. Additionally, perceived exercise benefit was positively correlated with physical activity level (*r* = 0.43), indicating that the higher the level of perceived exercise benefit, the more active the participants. Hierarchical regression analysis revealed that the main effects of cellphone usage and perceived exercise benefit explained 22% of the variance in physical activity level. After controlling for the main effect, the interaction term accounted for an additional 1% of the variance. The results of this study reveal a novel phenomenon—that the relationship between cellphone usage and physical activity is moderated by perceived exercise benefit. More specifically, students who perceived the benefits of exercise to be greater were more physically active.

In the current study, cellphone usage was negatively correlated with physical activity level, supporting the findings of prior research [42,43,44,45]. Physical exercise is negatively correlated with cellphone dependence and can be used to effectively predict cellphone dependence. The research also found that students with high cellphone dependence usually exercise less every day, and excessive use of cellphones may interfere with physical activity. In other words, improvement in physical education may be a highly feasible way to effectively solve the problem of cellphone dependence of university students and other young people around the world. Cellphone use is associated with lower physical activity. The rapid popularization of cellphones has prompted worries about the risks posed by cellphones toward health and quality of life [46]. Excessive cellphone use results in the majority of university students’ free time being spent on these devices. Therefore, cellphone usage appears to be a double-edged sword. Studies have indicated that university students treat cellphones more like entertainment tools rather than educational tools [44] and that cellphone usage affects physical activity [18]. The negative relationship between cellphone usage and physical activity discovered in the current study can be attributed to students spending too much time on the internet during their hours outside the classroom. Spending excessive time on online activities such as checking social media, playing games and messaging their friends decreases the likelihood that they spend time being physically active. Nevertheless, cellphones do have their benefits. When used appropriately, sports applications on smartphones can be used to support and promote physical activity [47,48], but this requires knowledge of what cellphone use is appropriate and an awareness of the benefits of sports to health.

The current study also discovered a moderating effect of perceived exercise benefit on the relationship between cellphone usage and physical activity level. Thus, physical activity guidelines and the benefits of exercise to health should be given more emphasis in university physical education curricula. The perception of students is a crucial factor influencing their physical activity level [49]. One study revealed that a combination of cognitive and behavioral strategies may help develop and strengthen the intention to be physically active and thus contribute greatly to people’s health and quality of life [8]. Physical education is the most common educational method employed to promote physical activity in schools and among adolescents [32]. Most unhealthy behaviors occur due to the lack of a relevant education curriculum [42]. Thus, physical education should offer more health-oriented courses and knowledge. In-depth understanding of the characteristics of physical activity (e.g., the perceived benefits of, and obstacles to, physical activity) could also improve the ability of physical education teachers to promote health, develop relevant interventions and, ultimately, improve the physical activity level of students outside classroom hours.

Physical education teachers should adopt a lifelong learning attitude and continually acquire knowledge and skills relevant to physical activity. Additionally, they should employ educational means to encourage students to improve the quantity and quality of their physical activity, disseminate knowledge on appropriate cellphone usage and warn students about the risks of physical inactivity. Modern cellphones contain numerous temptations to connect to the internet because their size means they can be carried everywhere and used at any time. In the context of physical education and public health, physical educators should play a driving role in promoting physical activity and health literacy. Additionally, physical educators should disseminate knowledge related to the effects of cellphone usage on physical activity or health through appropriate curriculum planning, encouraging students to participate in more sports activities.

### Limitations of the Study

This study was a cross-sectional study and could only confirm that significant relationships exist between the examined variables. The causal relationships between cellphone usage, perceived exercise benefit, and physical activity cannot be inferred from the results of the current study. To explore the causal relationships between these variables, an experimental study with appropriate time design would be required. Additionally, the current study sample comprised solely Taiwanese university students. Therefore, caution must be taken when generalizing the results to student populations of other countries or who receive different forms of education.

## 5. Conclusions

In conclusion, this study provides confirmatory evidence that significant correlations exist between cellphone use frequency, physical activity level and perceived exercise benefit. The study also discovered a moderating effect of perceived exercise benefit on the relationship between cellphone usage and physical activity level. Thus, physical activity guidelines and the benefits of exercise to health should be given more emphasis in university physical education curricula. Cellphone usage can predict physical activity level. Our data indicate that students with lower cellphone use are more physically active than their counterparts with greater cellphone use. Higher physical activity level can lead to more healthy behaviors and improvement of health status. Cellphone usage and perceived exercise benefit interacted with each other in predicting physical activity level in that perceived exercise benefit moderated the relationship between cellphone usage and physical activity level. Given that cellphones will continue to be omnipresent in universities, physical activity and health behaviors should be strengthened by incorporating the cognitive changes associated with cellphone usage and perceived exercise benefits into university physical education curricula.

## Figures and Tables

**Table 1:** Descriptive statistics and correlations between variables.

Variables	Cellphone Usage	Perceived Exercise Benefit	Physical Activity Level
Cellphone usage	1		−0.23 **
Perceived exercise benefit	−0.10 **	1	0.43 **
Physical activity level	−0.23 **	0.43 **	1
Mean	3.2	38.88	2.08
Standard deviation	1.08	7.16	0.67

** *p* < 0.01.

**Table 2:** Two-way interaction between cellphone usage and perceived exercise benefit.

Interaction	Physical Activity Level	
	ΔR^2^	β
Step1	0.22 ***	
(X1) Cellphone usage		−0.19 ***
(X2) Perceived exercise benefit		0.41 ***
Step 2	0.01 *	
(X1) Cellphone usage		−0.19 ***
(X2) Perceived exercise benefit		0.41 ***
X1X2 Interaction term		−0.07 *
Total R^2^	0.22 ***	
N	975	

* *p* < 0.05 *** *p* < 0.001.
